# Enhanced AmB Production in *Streptomyces nodosus* by Fermentation Regulation and Rational Combined Feeding Strategy

**DOI:** 10.3389/fbioe.2020.00597

**Published:** 2020-07-15

**Authors:** Bo Zhang, Yu-Han Zhang, Yu Chen, Kai Chen, Sheng-Xian Jiang, Kai Huang, Zhi-Qiang Liu, Yu-Guo Zheng

**Affiliations:** ^1^The National and Local Joint Engineering Research Center for Biomanufacturing of Chiral Chemicals, Zhejiang University of Technology, Hangzhou, China; ^2^Key Laboratory of Bioorganic Synthesis of Zhejiang Province, College of Biotechnology and Bioengineering, Zhejiang University of Technology, Hangzhou, China

**Keywords:** amphotericin B, differential metabolites, precursors, fermentation regulation, combined feeding strategy

## Abstract

Amphotericin B is a clinically important polyene macrolide antibiotic with a broad-spectrum antifungal activity. In this work, the addition of key precursors and differential metabolites, combined with staged fermentation process control strategies, was carried out to improve AmB production. Rationally designed addition strategies were proposed as follows: 4 mg/L isopropanol, 1 mM alanine, 1 g/L pyruvate, and 0.025 g/L nicotinamide were supplemented at 24 h. The AmB titer was ultimately enhanced to 6.63 g/L, with 28.5% increase in shake flasks fermentation. To further promote the biosynthesis of AmB, different glucose feeding strategies were investigated and the highest AmB titer (15.78 g/L) was obtained by constant speed fed-batch fermentation in a 5-L fermentor. Subsequently, compared with the batch fermentation (9.89 g/L), a novel combined feeding strategy was ultimately developed to improve the production of AmB by 85.9%, reaching 18.39 g/L that is the highest titer of AmB ever reported so far, in which the optimized components were fed at 24 h and the staged fermentation regulation strategies were used simultaneously. Moreover, the ratio of co-metabolite AmA decreased by 32.3%, from 3.1 to 2.1%. Through the detection of extracellular organic acids, the changes in α-ketoglutaric acid, pyruvate, and citric acid concentrations were identified as the most flexible metabolite nodes to further clarify the potential mechanism under different fermentation regulation strategies. These results demonstrated that the strategies above may provide new guidance for the industrial-scale production of AmB.

## Introduction

Amphotericin B (AmB), a polyene macrolide antibiotic predominantly produced by *Streptomyces nodosus* (Caffrey et al., 2001), is a broad-spectrum antifungal drug mainly used in the clinical treatment of deep fungal invasive infections (Vasquez et al., 2018). AmB exerts its fungicidal action by binding strongly to sterol components such as ergosterol and creating transmembrane channels through which essential cell constituents leak out (Shim et al., 2011). Moreover, the genes responsible for AmB biosynthesis have been cloned and sequenced (Caffrey et al., 2001; Sweeney et al., 2015). For the biosynthesis of AmB, the polyketide precursor is assembled from acetate and propionate units, further cyclization, hydroxylation at C8, mycosaminylation at C19, and finally the propionate-derived C41 methyl group is oxidized to form the exocyclic carboxyl group (Caffrey et al., 2001). The C28–C29 unsaturated bond in AmB completes the conjugation in the polyene region and increases the rigidity of the molecule (Volpon and Lancelin, 2002). When the ER5 domain in module AmphC plays roles, which is responsible for the reduction of the C28–C29 unsaturated bond during the biosynthesis of macrolides, co-metabolite amphotericin A (AmA, C28–C29 saturated bond), with low antifungal activity, is also produced simultaneously (Borgos et al., 2006), the result of which is that AmB and AmA are relatively difficult and time-consuming to separate. Although the clinical usage of AmB is limited because of its side effects, such as acute nephrotoxicity (Larabi et al., 2003), it is still irreplaceably applied as a useful antibiotic for more than 50 years for treating systemic fungal infections (Deray, 2002).

Due to the medicinal value and the clinical importance described above, related researches on amphotericin B and its derivatives are more in-depth and mature. Concurrently, many classical breeding methods of high-yield strains including fermentation development (Siddique et al., 2014; Ahsan et al., 2017; Ju et al., 2017; Zhao et al., 2017) and efficient bioprocesses (Santos-Aberturas et al., 2011; Yao et al., 2018) have been applied to increase the titer of several polyene macrolide antibiotics (Pereira et al., 2008; Zhang et al., 2017). Another important strategy was reported, such that short-chain fatty acid, lower alcohols, and some salt substances can be added as precursors of macrolide antibiotics to derive a high product titer (Jing et al., 2011). For example, the production of AmB-^3^H with a specific radioactivity was increased to 3.5 g/L by using acetate-^3^H as a precursor (Monji et al., 1976). In addition, the addition of nicotinic acid can improve the productivity of tacrolimus by 6-fold (Turło et al., 2012). Li et al. demonstrated that the addition of propanol is an effective strategy to increase natamycin yield through regulating the metabolite node and the pools of precursors (Li et al., 2014). Additionally, the sufficient availability of precursors and intermediates, which are supplied from primary metabolism, is a prerequisite for the biosynthesis and the productivity of different antibiotics (Huang et al., 2011; Xia et al., 2013; Qi et al., 2014a). Xia et al. tried to enhance FK506 titer in *Streptomyces tsukubaensis* by rational feeding strategies, which made it the first study to apply comparative metabolic profiling analysis to identify key metabolites as exogenous additives to promote FK506 production (Xia et al., 2013). In another study, 11 key metabolites that contributed most to metabolism differences and ascomycin biosynthesis were identified by partial least square (PLS) to latent structures discriminant analysis. Then, *n*-hexadecane, valine, and lysine, at optimum concentrations, were added at 24, 48, and 72 h. The ascomycin production was ultimately improved to 460 mg/L, a 53.3% enhancement compared with that obtained in the initial condition (Qi et al., 2014b).

Regulation of environmental conditions such as oxygen, pH, temperature, stirring speed, and medium supplementation with nutrients plays important roles in the biosynthesis of fermented products. Previous reports focus on strain improvement, optimization of culture conditions, and various fermentor operational processes to increase polyene macrolide antibiotic yield (Zhu et al., 2010; Chan et al., 2015). A selected strain capable of producing 4.5 g/L AmB in a complex medium was able to produce 3.5 g/L AmB in a defined medium by medium optimization (Linke et al., 1974). In addition, the influence of oxygen mass transfer intensity characterized by the rate of oxygen dissolution and the agitation rate, as well as the influence of dissolved oxygen concentration, on the process of AmB biosynthesis was studied to achieve a high-yield AmB (Vekshin and Malkov, 1989). Fermentation process regulation associated with key metabolism also contributes to the production of AmB (Huang et al., 2011; Fan et al., 2014; Qi et al., 2014b). Meanwhile, different feeding strategies were also applied to increase the production of polyene macrolide antibiotics (Choi et al., 2014; Liu et al., 2014; Mears et al., 2017).

**Figure 1:**
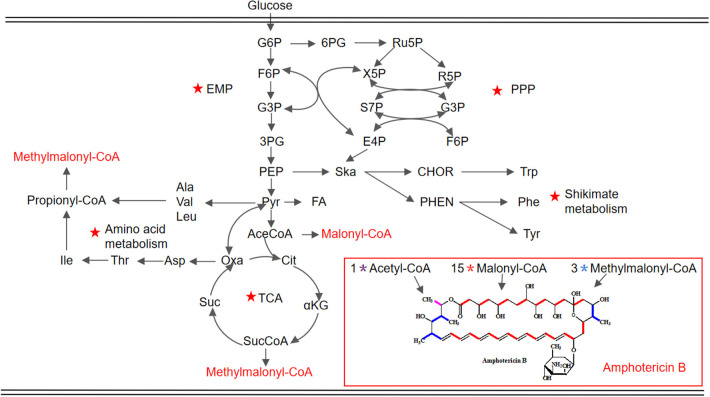
Schematic representation of the proposed metabolic pathways closely associated with AmB biosynthesis in *S. nodosus*. The key pathways are marked by red pentacles. EMP, Embden–Meyerhof–Parnas pathway; PPP, pentose phosphate pathway; TCA, tricarboxylic acid cycle. G6P, glucose 6-phosphate; F6P, fructose 6-phosphate; G3P, glyceraldehyde-3-phosphate; 3PG, 3-phospho-glycerate; PEP, phosphoenolpyruvate; Pyr, pyruvate; AceCoA, acetyl-CoA; Cit, citrate; αKG, α-ketoglutarate; SucCoA, succinyl-CoA; Suc, succinate; Oxa, oxaloacetate; Asp, aspartate; Thr, threonine; Ile, isoleucine; Ala, alanine; Val, valine; Leu, leucine; 6PG, 6-phospho-gluconate; Ru5P, ribulose 5-phosphate; X5P, xylulose 5-phosphate; R5P, ribose 5-phosphate; S7P, sedoheptulose 7-phosphate; E4P, erythrose 4-phosphate; Ska, shikimic acid; CHOR, chorismate; Trp, tryptophan; PHEN, prephenic acid; Phe, phenylalanine; Tyr, tyrosine.

**Figure 2:**
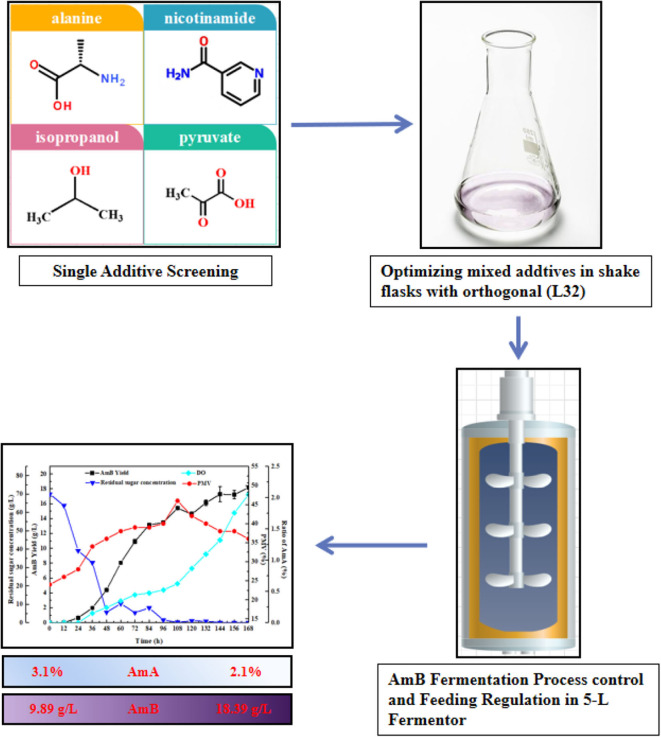
A flow chart for enhanced AmB production by fermentation strategy.

In order to enhance the productivity of AmB, it is critical to investigate more metabolic engineering or bioprocess automation strategies. New strategies in our lab were developed to optimize the biosynthesis of AmB based on in-depth analysis of key metabolites and pathways. Through overexpression genes involved in oxygen taking, precursor acquiring, and product exporting, the titer of AmB increased to 6.58 g/L, which is the highest titer of AmB in a shake flask as far as we know (Zhang et al., 2020). However, there has been no research to determine the key differential metabolites as additives to enhance AmB production. In this work, the addition of key precursors and metabolites closely associated with AmB biosynthesis was investigated. Ultimately, rational united feeding strategies of the targeted additives were established for an obvious improvement of AmB titer and low accumulation of AmA. In addition, different fermentation process regulation strategies were proposed toward providing an optimal environment for AmB-producing strains to enhance AmB production. Furthermore, this work presented the strategy of compound feeding fermentation process regulation combined with fed-batch fermentation strategy to increase AmB production. To more accurately analyze the changes in cellular metabolism, a schematic representation of the proposed metabolic pathways closely associated with AmB biosynthesis in *S. nodosus* is shown in detail in Figure 1. Meanwhile, the extracellular organic acid changes in response to the regulation strategies were analyzed, which indicated that more α-ketoglutarate and pyruvate consumed were converted into the key precursors of AmB biosynthesis. These results demonstrate that combined fed-batch fermentation strategy, which improved AmB titer with a 85.9% increase and the ratio of co-metabolite AmA decreased by 32.3%, has an industrial potential for the improvement of antibiotic production. Furthermore, a flow chart (Figure 2) was summarized to clearly display the entire experimental study process.

## Materials and Methods

### Microorganism and Growth Conditions

*S. nodosus* ZJB2016050 (CCTCC M2017426, China Center for Type Culture Collection, Wuhan, China) was screened as a high amphotericin B production strain in our lab, which was used in this study (Zhang et al., 2017).

The seed culture was grown in 250-mL flasks containing 50 mL of seed medium (tryptone 1.5%, yeast extract 1%, NaCl 0.5%, glucose 1%, and CaCO_3_ 0.1% w/v, with a pH of 7.0) on a rotary shaker. After incubation at 26°C and 200 rpm for 48 h, a 2-mL seed culture was used to inoculate 50 mL of fermentation medium (glucose 7%, cottonseed meal 2.5%, CaCO_3_ 0.9%, and K_2_HPO_4_ 0.01% w/v, with a pH of 7.0) for AmB production in a 500-mL shake flask at 26°C and 200 rpm for 4–6 days.

In a 5-L fermentor, 200 mL of seed mycelia suspension was inoculated into a 5-L fermentor for further fermentation. The fermentation medium was inoculated with seed culture to 10% (v/v) and cultivated at 26°C. The culture medium contained the same components as in the shake flask cultures. Fermentation was performed under the following conditions (primary condition): air flow, 6 L/min; agitation speed, 500 rpm; temperature, 26°C; pH under natural conditions; and working volume, 2 L. The process pH value was adjusted with 30% ammonia and acetic acid and maintained automatically throughout the cultures.

### Optimization of Compound Addition in Shake Flask

To improve the production of AmB, seven isometric short-chain fatty acids, lower alcohols, and salt substance with a final concentration (0–12 mg/L) were added as precursors in the medium at 0 h. After 108 h, the AmB titer was detected to investigate which was the optimal additive and the addition concentration of these precursors. Subsequently, addition time was optimized between 0 and 72 h. Furthermore, 15 key differential metabolites based on metabolomics analysis were screened as additives into the fermentation medium at 24 h (Zhang et al., 2020). The optimum addition volumes of these compounds from 0.01 to 2 g/L were investigated.

### Fed-Batch Fermentation Regulation in a 5-L Fermentor

Feeding in all fed-batch fermentations was initiated with 900 mL glucose solution (glucose concentration, 200 g/L), and the feeding substrates were pumped into the fermentor by using a computer-coupled peristaltic pump. First, with respect to the different pulse-feeding fermentation strategies including three ways (one-, two-, and three-pulse feeding), 200 mL glucose solution was pumped into the fermentor once. In constant speed fed-batch fermentation, 900 mL glucose solution was pumped into the fermentor at a feeding rate of 1.5 g L^−1^ h^−1^ when the residual glucose concentration in the culture medium was reduced to 30 g/L. In variable speed fed-batch fermentation, feeding of glucose solution commenced with 2.5 g L^−1^ h^−1^ feeding rate at 48 h after the fermentation began and altered the rate to 1.5 g L^−1^ h^−1^ until the fermentation time was 108 h. In constant residual glucose concentration fed-batch fermentation, the residual glucose concentration in the culture medium was maintained at 30 g/L by feeding glucose solution.

### Analytical Methods

Samples were taken from shake flasks and fermentor for analysis at regular intervals. The biomass concentration (PMV) was determined on the basis of its volume ratio between solid volume and total after centrifugation at 12,000 rpm for 10 min (Zou and Li, 2013). The glucose concentration was measured by an enzymatic reaction using glucose oxidase (using the glucose determination kit produced by Shanghai Rongsheng Biological Pharmaceutical Co., Ltd., Shanghai, China).

The concentration of AmB was determined by the high-performance liquid chromatography (HPLC) method. The dilution was separated by a Unitary C18 column (4.6 × 250 mm; 5-μm particle sizes) with the following mobile phase. The mobile phase was the organic phase which was formulated as follows: salt solution (1 L water containing 1.1 g EDTA·2Na and 4.1 g anhydrous sodium acetate):acetonitrile:methanol = 9:7:4 (by volume). At the time of treatment, 150 μL of the fermentation broth sample was added to 1,350 μL of dimethyl sulfoxide and extracted at 30°C for 20 min. Furthermore, after centrifugation, the supernatant was obtained. Then, 20 μL of the filtered sample, obtained by using a 0.22-μm filter, was injected into a C18 column and then eluted with the mobile phase at 1 mL/min and 25°C. AmA and AmB were then detected at 304 and 405 nm using a UV detector (Hitachi, Ltd., Japan), respectively. In addition, a commercial standard of AmB was obtained from Sigma-Aldrich (CAS: 1397-89-3) (Zhang et al., 2020).

The extracellular organic acids, including α-ketoglutaric acid, citric acid, succinic acid, formic acid, acetic acid, lactic acid, pyruvic acid, malonate, and oxalic acid, were detected by a Waters HPLC2489 series (Waters Corporation, Milford, MA, USA), equipped with an Aminex HPX-87H lon Exclusion column (300 × 7.8 mm). The column temperature was maintained at room temperature and the UV detector was set at 210 nm. The mobile phase, with a flow rate of 0.6 mL/min, contained 8 mM of sulfuric acid solution. The fermentation sample was centrifuged at 12,000 rpm for 5 min, then diluted to the appropriate multiple, and filtered through a 0.22-μm filter (Jinteng Experimental Equipment Co., Ltd., Tianjin, China). Each standard stock solution of the test organic acids was carefully prepared in distilled water (1 g/L) and stored at −4°C. The spiked standard solution was accordingly obtained by the addition of each aliquot of the stock solution above, involving 1 mL of each organic acid. Different volumes of the spiked standard solution were diluted into 1 mL of water for linear range determination.

### Orthogonal Design for United Additives and Statistical Analysis

The orthogonal design was applied to elucidate the optimal concentrations of the six most significant additives screened in the previous one-factor optimization experiment. As presented in Table S1, isopropanol (*X*_1_), serine (*X*_2_), alanine (*X*_3_), D-calcium pantothenate (*X*_4_), pyruvate (*X*_5_), and nicotinamide (*X*_6_) were prescribed as the six independent variables. According to the applied design, 32 combinations were executed, and their experimental results were fitted with a second-order polynomial equation of Equation (1) by a multiple regression technique.

(1) $$\begin{array}{l} Y=b_{0}+\sum b_{i}X_{i}+\sum b_{ii}{X^{2}}_{i}+\sum b_{ij}X_{i}X_{j} \end{array}$$

where *Y* is the dependent variable (AmB titer), *X*_i_ and *X*_j_ stand for the independent variables of the coded value, *b*_0_ is the regression coefficient at center point, *b*_*i*_ is the linear coefficient, *b*_*ii*_ is the quadratic coefficient, and *b*_ij_ (*i* ≠ *j*) is the interaction coefficient. The fitness of the second-order model was expressed by the regression coefficient *R*^2^, and the significant variables were considered based on the *F*-test and the *P*-value at a significance level of 5% (*P* < 0.05). All the experiments in this study were carried out in triplicate, and the experiment results were statistically analyzed by Minitab 17 software (Minitab Inc., State College, PA, USA).

## Results and Discussion

### Screening Key Precursors and Differential Metabolites for AmB Biosynthesis

**Figure 3:**
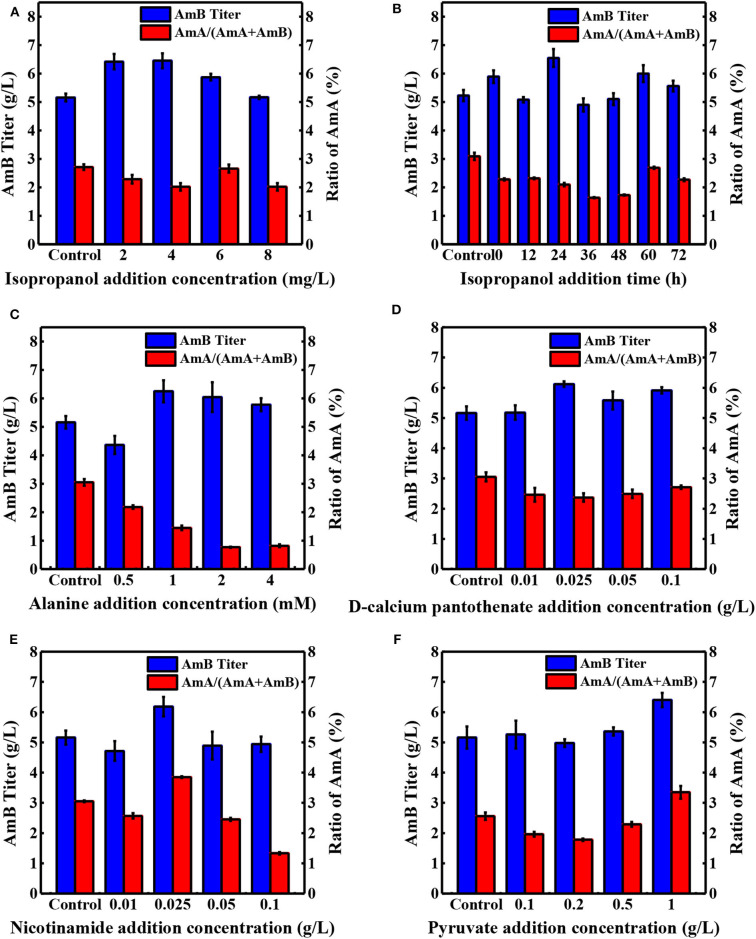
Effects of different concentrations of additives on the production of AmA and AmB. **(A)** Isopropanol, **(C)** alanine, **(D)** D-calcium pantothenate, **(E)** nicotinamide, and **(F)** pyruvate at 108 h. **(B)** The optimal addition time was determined with isopropanol. The blue and red bar charts represent the concentration of AmB in the sample and the proportion of by-product AmA in the total amount of AmA and AmB, respectively. Each value is calculated from at least triplicate cultures.

Precursor supplement and key metabolite addition significantly influence the secondary metabolite production (Mao et al., 2009; Xia et al., 2013). As shown in Table 1, seven precursors and 15 key differential metabolites were determined as additives and tested in shake flask fermentation based on the biosynthesis of AmB and the metabolite analysis (Zhang et al., 2020). When 10 mg/L sodium propionate was supplemented, the highest AmB titer (6.93 g/L) was achieved, with 34% increase. However, the content of the by-product AmA increased to 8%. The structure of AmB suggests that the polyketide precursor of AmB is assembled from acetate and propionate units (Caffrey et al., 2001). Obviously, the high level of sodium propionate can offer more propionate units to incorporate into AmB and AmA. In a previous study, Potvin et al. found that propanol had positive effects on erythromycin production (Potvin and Péringer, 1993). Interestingly, we hardly observed a significant advantage from increasing the propanol levels in our study. Combined with pathways of the two macrolide antibiotic biosynthesis (Staunton and Wilkinson, 1997; Caffrey et al., 2001), it was inferred that different producing strains resulted in the dissenting ability of propanol to inhibit cell growth, leading to different final target product yields. With respect to isopropanol, the results indicated that the AmB titer (6.45 g/L) was promoted by 25% and the ratio of AmA decreased to 2% (Figure 3A). The low concentration of isopropanol in the medium could slightly enhance the structural stability of the protein to further improve the AmB titer (Cinelli et al., 1997). Besides that, the weakened affinity between the NADPH cofactor and the ER5 domain likely explains why isopropanol is a better additive for reducing the AmA production, which apparently arises from the failure of enoyl reductase to function during the antibiotic biosynthesis (Borgos et al., 2006). Furthermore, the precursor addition time was optimized for isopropanol (0, 12, 24, 36, 48, 60, and 72 h), and the most effective addition time was 24 h, as depicted in Figure 3B. Finally, isopropanol and 24 h were determined as the most effective precursor and addition time, respectively, for the following experiments.

**Table 1:** Effects of precursor and key metabolite addition for AmB production.

**Compounds**	**Optimized final concentration**	**Ratio of AmA (%)**	**AmB production (g/L)**
Control	-	3.05 ± 0.24	5.16 ± 0.14
Sodium propionate	8 mg/L	7.86 ± 0.85	6.93 ± 0.27
Calcium acetate	10 mg/L	5.13 ± 0.34	6.04 ± 0.03
Acetic acid	0.2 g/L	1.90 ± 0.08	5.14 ± 0.10
Ethanol	2% (v/v)	3.10 ± 0.39	3.37 ± 0.54
Propanol	6 mg/L	4.01 ± 0.27	5.65 ± 0.52
Isopropanol	4 mg/L	2.14 ± 0.28	6.45 ± 0.06
Glycerol	0.05 g/L	4.25 ± 0.18	6.04 ± 0.36
Mannose	0.05 g/L	3.67 ± 0.18	5.15 ± 0.17
Trehalose	0.025 g/L	2.75 ± 0.14	5.26 ± 0.54
Alanine	1 mM	1.86 ± 0.09	6.25 ± 0.09
Serine	4 mM	4.76 ± 0.02	6.90 ± 0.74
Glycine	0.5 mM	3.92 ± 0.08	5.74 ± 0.18
D-Calcium pantothenate	0.025 g/L	2.37 ± 0.14	6.12 ± 0.09
Lecithin	0.1 g/L	5.67 ± 0.63	4.46 ± 0.15
Calcium phosphate	0.01 g/L	2.89 ± 0.06	5.26 ± 0.17
Inosine	0.025 g/L	3.46 ± 0.12	5.36 ± 0.24
Nicotinamide	0.025 g/L	3.84 ± 0.03	6.18 ± 0.32
Pyruvate	1 g/L	3.35 ± 0.08	6.40 ± 0.24
Succinic acid	0.025 g/L	3.13 ± 0.05	5.26 ± 0.54
Citric acid	0.5 g/L	1.59 ± 0.08	5.26 ± 0.15
Shikimic acid	0.25 g/L	2.20 ± 0.03	5.18 ± 0.09
Lactic acid	0.25 g/L	2.38 ± 0.13	5.40 ± 0.39

Metabolites were supplemented into the fermentation medium to further improve AmB production and the control group represented the contrast without addition. Each value was calculated from at least triplicate cultures. Ratio of AmA = [AmA / (AmA + AmB)] * 100%. For the addition of glycerol, we performed our study based on Juan Francisco Martin's research. The group found that glycerol indeed increased the yields of several amphotericin-related polyenes (Martín and Aparicio, 2009).

The central carbon metabolism pathways (Embden–Meyerhof–Parnas pathway, EMP; pentose phosphate pathway, PPP; and tricarboxylic acid cycle, TCA) could provide essential energy to promote the cell growth and the key intermetabolites (Mattozzi M. de la Peña et al., 2010; Lu et al., 2013; Ng et al., 2014). Besides supporting protein synthesis and cell growth, amino acid catabolism was confirmed as an important source of polyketide precursors (Tang et al., 1994). In this study, to investigate the influence of intermetabolites in the EMP, TCA, and PPP pathways and in amino acid catabolism on AmB production (Figure 1), various compounds were added into the medium with different concentrations at 24 h (Table 1 and Figure 3). Compared with the control, the ratio of AmA with pyruvate addition increased from 3.1 to 3.4%, and the production of AmB (6.40 g/L) was highest with a 24.1% improvement, which indicated that extra pyruvate stimulated the conversion of more pyruvate into acetyl-CoA and further into methylmalonyl-CoA, a precursor of AmB (Xia et al., 2013). It was worth noting that the supplementation of 0.025 g/L nicotinamide resulted in 6.18 g/L of AmB titer, which displayed 19.8% improvement (Figure 3E). Besides that, the ratio of AmA reached 3.8%. This may due to the reason that nicotinamide could offer more reducing power, in the form of NADPH, required for the biosynthesis of polyketides (Borgos et al., 2006; Turło et al., 2012). Furthermore, sufficient NADPH cofactor guaranteed the catalytic activity of ER5 domain to produce more AmA (Borgos et al., 2006). However, the AmB yield failed to be strengthened by the addition of citric acid and succinic acid in the TCA pathway.

When different amino acids were ingested into the medium at 24 h, 4 mM serine addition showed the highest titer increase (34.2%), followed by alanine (28.4%), and glycine (10%). Based on our previous metabolite analysis, 5,6,7,8-tetrahydrofolate (THF), 5,10-methyltetrahydrofolate, and 5-formiminotetrahydrofolate were selected as several metabolites contributing the most into the groups' discrimination (Zhang et al., 2020). The amphotericin yields were increased by serine and glycine. These amino acids contribute one-carbon units into the THF pool via the serine hydroxymethyl transferase reaction and the glycine cleavage system. The availability of methyl-THF could stimulate the methionine salvage pathway and boost intracellular levels of SAM, which is known to elevate polyketide yields in other streptomycetes by increasing expression of polyketide biosynthetic genes (Kim et al., 2003; Okamoto et al., 2003). The addition of alanine could play important roles in the AmB production process by significantly enhancing acetyl-CoA and methylmalonyl-CoA for AmB biosynthesis (Liu et al., 2018). Meanwhile, the ratio of AmA with alanine addition obviously decreased to 1.9%. For unknown reasons, the enoyl reduction catalyzed by module 5 of the AmphC PKS is apparently less efficient after the addition of alanine, resulting in a decrease in the final output of AmA. Moreover, the ratio of AmA decreased to 2.4% and AmB production increased by 18.6% in the group supplemented with 0.025 g/L D-calcium pantothenate (Figure 3D). Undoubtedly, higher levels of D-calcium pantothenate could have a greater contribution to the biosynthesis of AmB. In the previous research, the positive effect of aromatic amino acids and shikimic acid supplementation on rapamycin production was reported (Zhao et al., 2013). However, it is in this study that the addition of shikimic acid failed to promote the production of AmB.

### Improvement of AmB Production Based on Rational United Addition Strategy

In order to further strengthen the biosynthesis of AmB, several additives were fed at 24 h by applying orthogonal experimental design L32 (Table S1). The effects and the regression coefficients of each factor in Table 3 were obtained by analyzing the experimental results with Minitab 17 software. The *R*^2^ value was 0.917, indicating that 91.7% of the variability in the response could be explained by the model, and alanine (*X*_3_, *P* < 0.05) was screened to be the most major factor that influences AmB production. Non-significant factors *X*_2_ (serine) and *X*_4_ (D-calcium pantothenate) should be taken at low levels because of negative effects. In addition, *X*_1_ (isopropanol), *X*_5_ (pyruvate), and *X*_6_ (nicotinamide) should be taken at high levels due to the positive effects, which was consistent with previous experimental results. The obtained regression curve is as follows:

(2) $$\begin{array}{l} Y=5.2663+0.0787X_{1}-0.0787X_{1}^{2}-0.0087X_{2}-0.0087X_{2}^{2}\\ \;-0.2122X_{3}+0.2122X_{3}^{2}-0.0608X_{4}-0.0608X_{4}^{2}\\ \;-0.0648X_{5}+0.0648X_{5}^{2}+0.0225X_{6}+0.0225X_{6}^{2} \end{array}$$

**Figure 4:**
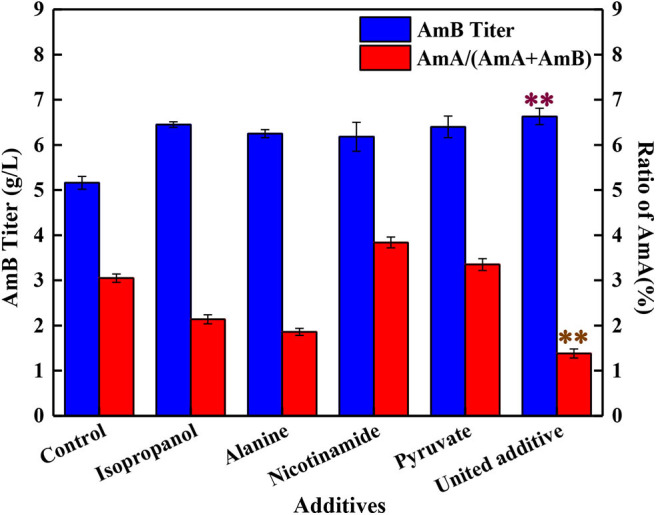
Effects of different addition strategies on the production of AmA and AmB, including the separate addition of isopropanol, alanine, nicotinamide, and pyruvate and the supplementation of united additives. Double asterisk symbols represent the optimum additive feeding strategy. The values shown represent the means of three independent experiments and the error bars represent the standard deviations of three values.

Compared with the control groups, when 4 mg/L isopropanol, 1 mM alanine, 1 g/L pyruvate, and 0.025 g/L nicotinamide were added altogether, the AmB titer was improved to 6.63 g/L, with 28.5% enhancement, which was higher than adding any kind of additive separately (Figure 4). Meanwhile, the ratio of AmA decreased to 1.4%, and the biomass ranged from 22% to 26% by the united addition strategy. Taking into consideration the analysis of single compound addition above, a positive promotion was observed between AmB biosynthesis and pyruvate (or nicotinamide) addition. Meanwhile, it was inferred that isopropanol and alanine addition might be treated as a limiting factor for AmA biosynthesis in united addition strategy to decrease the AmA content.

### Optimal Fermentation Process Regulation in a 5-L Fermentor

On the basis of the experiments in shake flasks, AmB fermentation was scaled up to a 5-L fermentor. The production of AmB was cultivated at natural pH, 26°C, and 500 rpm, increasing from 5.16 g/L in the shake flask to 9.89 g/L in the 5-L fermentor (increased by 91.7%) without a significant change on the AmA content (3.1%). The initial glucose concentration and the agitation rates were investigated firstly. As shown by the results, the agitation rate at 500 rpm and the initial glucose concentration of 70 g/L (Tables S2, S3; Figures S1, S2) were most conducive to cell growth and AmB production. Then, process regulation was conducted to improve the fermentation performance, including pH, temperature, and dissolved oxygen (DO), on a 5-L fermentor.

**Figure 5:**
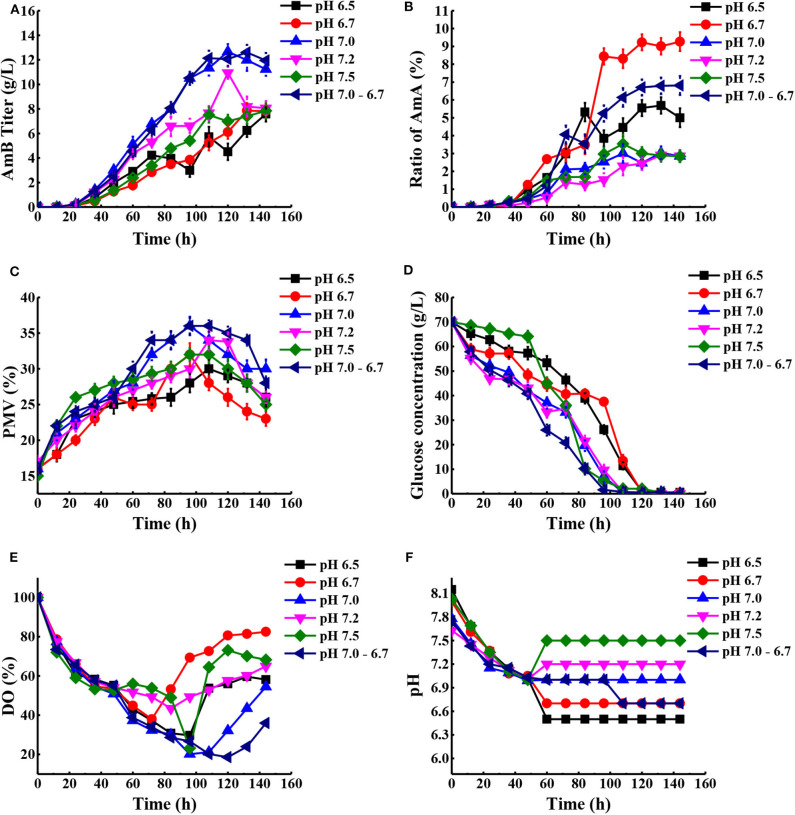
Comparison of fermentation performances under different pH conditions. **(A)** AmB titer with time, **(B)** ratio of AmA with time, **(C)** cell growth, **(D)** consumption of glucose, **(E)** change of DO levels, and **(F)** different pH conditions in the process. Each value represents the mean of three separate determinations with standard deviation.

According to previous research, the optimum pH value for the synthesized *Streptomyces* antibiotic was maintained at 6.0–7.8 (Kluepfel et al., 1986; Chungool et al., 2007; Li et al., 2008). In this work, the pH staged control strategy was implemented. The process pH was not controlled until the pH decreased to 7.0 and then maintained at 6.5, 6.7, 7.0, 7.2, and 7.5 to the end of the fermentation, respectively (Figure 5). As observed in Table 2, the highest AmB titer increased up to 12.66 g/L, with 28.4% improvement, and the maximum biomass reached 36% at pH 7.0. At the same time, the productivity of AmB was improved from 0.069 to 0.088 g L^−1^ h^−1^ at 144 h. Moreover, the ratio of AmA for pH at 6.5, 6.7, 7.0, 7.2, and 7.5 was 5.7, 9.3, 3.0, 2.9, and 3.5%, respectively. These results indicated that the optimal pH value for low AmA was around 7.0–7.2. Based on the result under the pH-uncontrolled condition, AmB kept a rapid synthesis status, with the pH ranging from 6.7 to 6.9, when the cells entered into the stationary phase. Furthermore, since the optimum pH for cell growth and product formation is possibly different, a new three-stage pH control strategy (pH: 0–48 h in nature status, 48–108 h at 7.0, and 108–144 h at 6.7) was proposed for further enhancement of AmB production. Here the two strategies had similar performance on the AmB synthesis rate and biomass (36%). However, pH 7.0 showed an advantage over pH 7.0–6.7 in terms of glucose consumption rate and AmA biosynthesis (Figures 5B,D). The result demonstrated that the pH 7.0 control strategy was more effective than the three-stage pH 7.0–6.7 control strategy for AmB production (Table 2).

**Table 2:** Batch fermentation parameters for AmB production under different pH, temperature, and dissolved oxygen conditions.

	**Period (h)**	**PMV (%)**	**Ratio of AmA (%)**	**Y_**AMB/glucose**_ (g/g)**	**Production of AmB (g/L)**	**AmB productivity (g L^**−1**^ h^**−1**^)**
**pH**						
Uncontrolled	144	32	3.1	0.141	9.89	0.069
6.5	144	30	5.7	0.108	7.59	0.053
6.7	144	32	9.3	0.112	7.82	0.054
7.0	144	36	3.0	0.181	12.66	0.088
7.2	144	34	2.9	0.156	10.94	0.076
7.5	144	32	3.5	0.156	10.93	0.076
7.0–6.7	144	36	6.8	0.180	12.60	0.088
**Temperature (°C)**						
24	144	28	4.9	0.101	7.07	0.049
26	144	32	3.1	0.149	10.13	0.072
28	144	30	3.2	0.139	9.74	0.068
30	144	34	3.8	0.157	11.01	0.076
32	144	35	4.2	0.128	8.95	0.062
30–26	144	34	4.5	0.168	11.79	0.082
**Dissolved oxygen (%)**						
20	144	37	2.8	0.162	11.28	0.078
30	144	34	2.7	0.148	10.39	0.072
40	144	33	2.5	0.146	10.21	0.071

*AmB, amphotericin B; PMV, biomass concentration; Y_AMB/glucose_, conversion yield of glucose to AmB*.

*Each value is calculated from at least triplicate cultures*.

Additionally, a previous report indicated that the organic acids may be reused as a potent carbon substrate and returned to the cell (Corsini and Tourneau, 1973). In order to understand the potential metabolic status within the strain, the concentration of extracellular organic acids under different pH conditions at 48, 72, 120, and 144 h was detected by HPLC (Table S4). Combined with the proposed metabolic pathways closely associated with AmB biosynthesis in Figure 1, the change of extracellular organic acids was analyzed in detail. Compared with two other pH strategies, the concentration of α-ketoglutarate and citric acid at pH 7.0 was significantly highest at about 1.9 and 3.2 g/L during the whole stage of fermentation, respectively. α-Ketoglutarate can be converted to methylmalonyl-CoA, through succinyl-CoA, to participate in AmB production. Based on the carbon metabolic network of *S. tsukubaensis*, pyruvate flux can be channeled to lactate, acetate, and the TCA pathway *via* acetyl-CoA (Xia et al., 2013). At pH 7.0 and 7.0–6.7, the pyruvate concentration was always maintained at a low level (<0.4 g/L), compared with that at pH 6.7. We speculated that more pyruvate consumed may enter into AmB biosynthesis to improve the AmB titer. In addition, a remarkable enhancement of formic acid concentration at pH 7.0 was observed during the major secondary metabolite biosynthesis phase (>96 h), and it was verified at a lower level, both at pH 6.7 and 7.0–6.7. When oxygen supply was insufficient in the cell, pyruvate could be reduced to lactic acid or acetic acid (Qi et al., 2014b). In brief, the lowest concentration of acetic acid (2.9 g/L) and lactic acid (<0.1 g/L) during fermentation under the pH 7.0 control strategy suggested that more pyruvate entered into the TCA pathway *via* acetyl-CoA and the primary metabolism of the cells was more active in this condition. These results indicated that the pH 7.0 control strategy can successfully increase the cell metabolism activity and extend the AmB synthesis period for higher AmB production.

**Figure 6:**
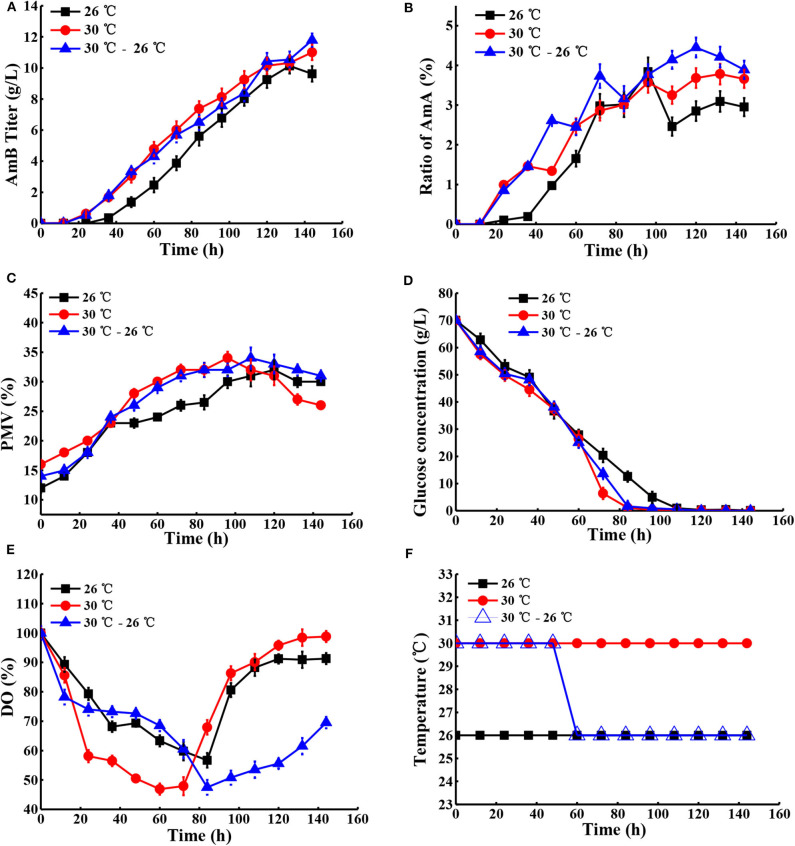
Comparison of fermentation performances at different temperatures. **(A)** AmB titer with time, **(B)** ratio of AmA with time, **(C)** cell growth, **(D)** consumption of glucose, **(E)** change of DO levels, and **(F)** different temperature conditions in the fermentation process. Each value represents the mean of three separate determinations with standard deviation.

Next, the effect of temperature, ranging from 24 to 30°C, on cell growth and AmB production was examined under natural pH and DO conditions. As shown in Table 2, the maximum biomass at different temperatures of 24, 26, 28, 30, and 32°C was 28, 32, 30, 34, and 35%, respectively, which indicated that high temperature (30°C) with the highest AmB titer (11.01 g/L) could accelerate cell growth. In particular, we postulated that some enzymes associated with AmB biosynthesis, such as ketoreductase, cytochrome P450 enzymes, etc., may have higher optimum temperatures and promoted product synthesis by affecting the activity of related enzymes, which was consistent with other studies (Peng et al., 2010; Li et al., 2017). Furthermore, when the temperature exceeded 30°C, the cell growth was continuous, but the biosynthesis of AmB was essentially inhibited (Table 2). Moreover, it was noted that the ratio of AmA was accompanied by increased temperature. In general, the temperatures required for microbial growth and product formation are different in most cases (Peng et al., 2010; Wei et al., 2012; Zhu and Fang, 2013; Tang et al., 2018). A new two-stage temperature control strategy was performed in our study. Under this strategy, the temperature was kept at 30°C for the first 48 h to accelerate the cell adaptation period in the early stage and then switched to 26°C to maintain high AmB accumulation in the later fermentation process. The final production of AmB obtained was 11.79 g/L, with 19.2% increase and productivity of 0.082 g L^−1^ h^−1^ (Figure 6). The maximum biomass (34%) was reached earlier (96 h) at 30°C than the situation at 30–26°C (34%) and was followed by a significant decrease, which was supposed to result in the AmB biosynthesis entering into a slow phase. Under the two-stage temperature control strategy, the highest AmA content (4.5%) was achieved and exhibited a continuous increase before 120 h, whereas at 30°C, the ratio of AmA (3.8%) entered into a plateau in the late phase (>120 h). We speculated that the promoting role of temperature change strategy on AmA formation may more effectively activate the enoyl reductase domain in module 5 of the AmphC PKS to promote AmA biosynthesis (Bruheim et al., 2004). These results indicated that the two-stage temperature control strategy contributed to AmB accumulation, with a small increase of AmA.

To understand how temperature change stimulated the secondary metabolite synthesis of *S. nodosus*, the change in extracellular organic acids was analyzed (Table S5). Here the α-ketoglutarate concentration gradually increased before 72 h under the two-stage temperature control strategy and, subsequently, was kept at a stable level of about 4.0 g/L, while with the other temperature control strategies, it continued to increase. In a previous report, it was proven by PLS analysis that citric acid and α-ketoglutaric acid were positively correlated to the accumulation of rapamycin (one of the polyketides) (Zhao et al., 2013). In view of the effect of the key metabolites in the TCA pathway on AmB biosynthesis (Figure 1), we speculated that more α-ketoglutarate was finally transformed into methylmalonyl-CoA, leading to its lower level, which may be another key factor for more AmB biosynthesis. According to Table S5, it showed that numerous pyruvate production (1.9 g/L) was possibly favorable for AcCoA accumulation before 72 h, and then it declined markedly (0.6 g/L) under the two-stage temperature control strategy. With respect to the change of succinic acid at different times, it was possible to further explain why the AmB production was enhanced markedly. The reason was that the two-stage temperature control strategy might lead to an increase of succinate, and succinate inhibits the activity of α-ketoglutarate dehydrogenase, which makes α-ketoglutarate increase in a short time and then it reduces, indirectly leading to increasing AcCoA and improving the AmB production (Li et al., 2014). Compared with the 26°C control strategy and the 30°C control strategy, the concentration of acetic acid experienced a significant change and reached the lowest level of 3.0 g/L in the final fermentation.

DO also has a close connection with various antibiotic production during the fermentation process (Dzhavakhiya et al., 2016). In this study, the effect of different DO levels was examined by using a set of experiments, with the pH adjusted to ~7.0 at 26°C. As shown in Table 2, DO regulation displayed prominent performance on AmB production. When DO was controlled at 20%, the highest AmB titer of 11.28 g/L was achieved, which was 14.1% higher than that of the uncontrolled condition. However, AmB production of about 10.39 and 10.21 g/L was achieved when DO was controlled at 30 and 40%, respectively. Meanwhile, in Table 2, it is indicated that the low DO level had positive effects on cell growth, and AmB production was limited by a higher DO concentration. The integrated results also revealed that the biomass reached the maximum (37%) at 20% DO, showing that AmB production was highly dependent on cell growth with DO improvement (<40%), which is in agreement with the previous report (Zhang et al., 2018). In addition, the DO control strategy (20, 30, and 40%) slightly decreased the ratio of AmA to 2.8, 2.7, and 2.5%, respectively. Here, compared with the control, the oxidation efficiency of NADH through the fermentation pathway is lower under oxygen-limited conditions, leading to the increment of intracellular NADH/NAD^+^ ratio, which caused the more active ER5 domain to increase the AmA content (Zaunmuller et al., 2006). The changes in extracellular organic acids were detected under different DO regulation strategies. As described in Table S5, no significant difference in the abovementioned metabolite concentration was found among the different DO conditions.

### Enhanced AmB Production by Different Fed-Batch Fermentation

Fed-batch fermentation technology allows the acquisition of high cell densities through the continuous supply of fresh nutrients. Many important additives, enough carbon and nitrogen sources fed into the medium, were shown to play an important role in AmB biosynthesis. Based on the results in the shake flasks, the additive feeding strategy was amplified to a 5-L fermentor. The results showed that the effect of additives in the shake flasks and the 5-L fermentor was basically consistent, except for the situation when adding nicotinamide individually, where the AmB titer was of a lower level (data shown in Figure S3) because the oxygen uptake rate, the oxygen transfer coefficient (*K*_La_), and the biomass obtained in the fermentor were quite different from those obtained in the shake flask (Çalik et al., 2004). However, the lack of carbon source in the fermentation process will also lead to insufficient cell growth and low AmB titer. The cell growth rate in fed-batch fermentation can be controlled by the application of growth-limiting feeding strategies, which is generally superior to the batch fermentation process (Liu et al., 2014). A fast-acting carbon source glucose was decided as the only carbon source in this research which might affect the NADPH level in the cells, based on earlier studies (Borgos et al., 2006), so the effects of different glucose feeding regulations on AmB fermentation production were further explored.

Based on the above analysis, several fed-batch fermentation strategies were successfully conducted to produce AmB with a higher concentration and control the cells under the optimal condition in a 5-L fermentor. The results are summarized in Table 4. Compared with the three different pulse-feeding fed-batch (PFB) strategies, one-time PFB fermentation had the highest AmB titer (11.42 g/L) and biomass (32.5%) and the lowest AmA content (1.9%). Repeatedly instantaneous high concentrations of glucose strengthened the substrate inhibition, which was not conducive to cell growth and AmB accumulation. Correspondingly, the level of AmA also increased with the number of glucose pulses. In general, we concluded that the activity of the AmA-synthesizing enzymes may be repressed by high glucose (Borgos et al., 2006). According to Table 3, the constant residual glucose concentration fed-batch (CGFB) strategy had a higher AmB concentration (14.51 g/L), with productivity of 0.086 g L^−1^ h^−1^, while the constant speed fed-batch (CSFB) strategy had the best AmB outcome (15.78 g/L), with productivity of 0.094 g L^−1^ h^−1^. The biomass under the CGFB and the CSFB strategies were 44.5 and 40.5%, respectively, which indicated that the AmB production and the cell growth were not necessarily synchronous when the biomass exceeds 40%. Due to the different glucose consumption rates in several fermentation stages, we speculated that the CSFB strategy successfully controlled the glucose concentration at a low level to minimize the substrate inhibition to make the cells in optimal condition and consequently resulted in the highest titer and productivity of AmB. Additionally, the effect of variable speed fed-batch strategy was examined to support greater AmB production (13.94 g/L), with the highest biomass (49.6%), which further showed that cell growth was not necessarily positively correlated with production. However, the ratio of the by-product AmA was correspondingly increased by 3–10%, compared with the batch fermentation, just by the feeding regulation strategies. This is because the efficiency of the ER5 domain, relative to AmA synthesis, can be influenced by carbon source availability in the amphotericin producer (Borgos et al., 2006). These results suggested that reasonably controlling the concentration of glucose in the fermentation process is an effective way to keep the cells in good growth and guarantee high AmB production status.

**Table 3:** Analysis of the effects of factors in orthogonal experiments.

**Term**	**Coefficient**	**SE coefficient**	***T*-value**	***P*-value**	**Variance inflation factor**
Constant	5.2663	0.0722	72.91	0.000	1.00
*X*_1_	0.0787	0.0722	1.09	0.286	1.00
*X*_2_	−0.0648	0.0722	−0.90	0.378	1.00
*X*_3_	−0.2122	0.0722	−2.94	0.007	1.00
*X*_4_	−0.0608	0.0722	−0.84	0.408	1.00
*X*_5_	0.0087	0.0722	0.12	0.906	1.00
*X*_6_	0.0225	0.0722	0.31	0.758	1.00

*X_1_, isopropanol; X_2_, serine; X_3_, alanine; X_4_, D-calcium pantothenate; X_5_, pyruvate; X_6_, nicotinamide; P ≤ 0.0001, highly significant; P ≤ 0.05, significant; P > 0.05, not significant*.

**Table 4:** Comparison of AmB production by different feeding strategies in fed-batch fermentation.

**Fermentation type**	**PMV (%)**	**Ratio of AmA (%)**	**Titer (g/L)**	**Yield (g/g)**	**Productivity (g L^**−1**^ h^**−1**^)**
Batch culture	34	3.0	10.12	0.146	0.070
One-time pulsed feeding fed-batch (84 h)	32.5	1.9	11.63	0.111	0.075
Two-time pulsed feeding fed-batch	27.9	4.4	9.68	0.077	0.058
Three-time pulsed feeding fed-batch	26.4	3.8	8.78	0.070	0.052
Constant speed fed-batch	40.6	7.1	15.78	0.101	0.094
Variable speed fed-batch	49.6	9.8	13.94	0.105	0.083
Constant residual glucose concentration fed-batch	44.5	8.8	14.51	0.109	0.086

Pulsed feeding fed-batch including one-, two-, and three-time pulsed feeding fed-batch. Ratio of AmA = [AmA / (AmA + AmB)] * 100%.

*PMV, biomass concentration*.

### Enhanced AmB Production Under Combined Feeding Strategy

Biosynthesis limitation by the lack of some key precursors and unfavorable environment was probably the extra reason for the restricted AmB production. To address these problems, combined feeding (CF) strategy was taken into consideration. To decrease the ratio of AmA and further increase the biosynthesis of AmB, the optimal mixed additives were pumped into the fermentor at 24 h after fermentation. When glucose and the optimal mixed additives were sufficiently available under a constant fed-batch combined with optimal staged fermentation regulation strategy, the cell growth (42%) was optimal, which resulted in maximal 18.39 g/L AmB production, with 85.9% increase, and the productivity of AmB and the AmA content was 0.11 g L^−1^ h^−1^ and 2.05%, respectively.

**Figure 7:**
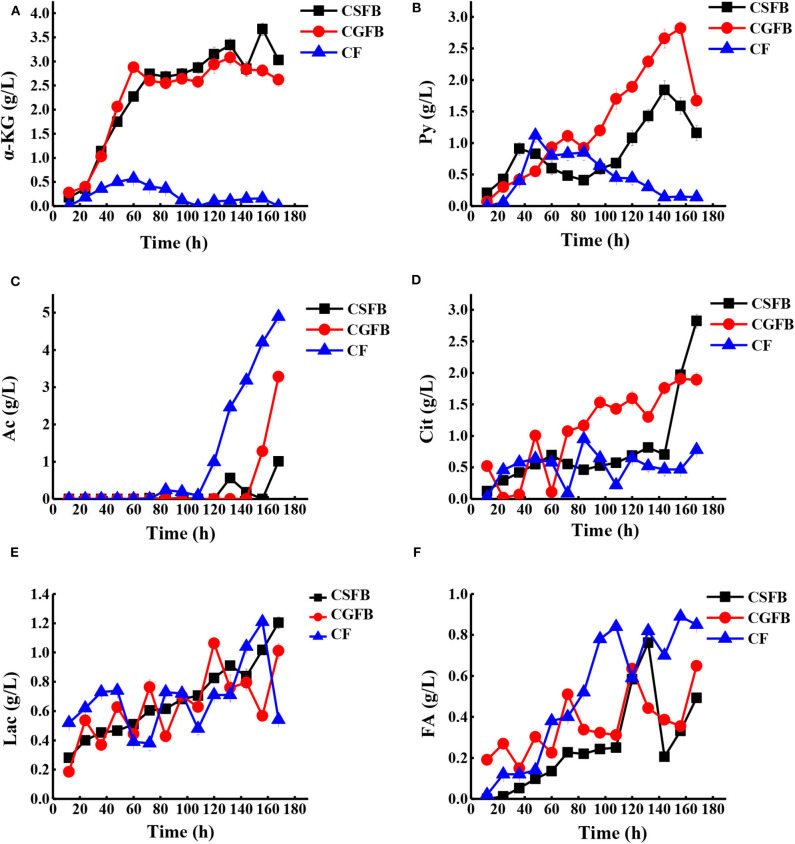
The time curves of organic acid concentrations with *S. nodosus* cultivated in a 5-L fermentor under different feeding strategies. **(A)** α-Ketoglutaric acid, **(B)** pyruvic acid, **(C)** acetic acid, **(D)** citric acid, **(E)** lactic acid, and **(F)** formic acid. The black symbol represents constant speed fed-batch strategy, the red symbol represents constant residual glucose concentration fed-batch, and the blue symbol represents combined feeding strategy.

The concentration of various extracellular organic acids (Figure 7) was detected to reveal the effect of combined feeding strategy on cell metabolism, and according to the metabolic pathways in Figure 1, we further illustrated why the maximal production of AmB has been achieved. α-Ketoglutarate and pyruvate in the central carbon metabolism gradually increased in the primary fermentation stage and were utilized quickly after 156 h under different feeding strategies (Figures 7A,B). Among them, the concentration of α-ketoglutarate and pyruvate was maintained below 0.5 and 1.0 g/L, respectively, under the CF strategy, while they peaked at 3.7 and 3.0 g/L and at 2.8 and 1.9 g/L under the CSFB and the CGFB strategies, respectively. It could be reasonably assumed that more extracellular α-ketoglutarate and pyruvate might be returned to the cells and converted to acetyl-CoA and then further to malonyl-CoA and methylmalonyl-CoA, a direct and important precursor of AmB. The tendency toward by-products (acetic acid, citric acid, lactic acid, and formic acid) is shown in Figures 7C–F. Compared with two other strategies, the acetic acid concentration arrived at the lowest level of 5.0 g/L under the CF strategy. The accumulation of acetic acid will inhibit cell growth and the expression of foreign proteins to further influence the biosynthesis of AmB. Under the CF strategy, the carbon metabolism flow in the EMP pathway is more restricted, thereby reducing the accumulation of acetic acid. In addition, the concentration of lactic acid experienced a large change, except that the lactic acid of the CSFB strategy was only gradually enhanced during fermentation. Specifically, citric acid can significantly affect cell growth and product accumulation (Zhao et al., 2013). The high level of citric acid may produce adverse effects on cell growth, leading to a decrease of cell metabolism and even death of the cell. More extracellular citric acid was transported back into the cells to participate in the TCA pathway under the CF strategy. Finally, the low levels of extracellular citric acid content in the whole AmB production process guaranteed a favorable environment for rapid and continuous AmB biosynthesis (Figure 7D). As described in Figure 7F, it was proven in this study that AmB production was positively associated with high formic acid concentration. After glucose produced pyruvate through the EMP pathway, pyruvate will be cleaved to produce acetyl-CoA and formic acid. The high concentration of formic acid demonstrated that more acetyl-CoA can participate in the biosynthesis of AmB. The results of the organic acid analysis indicated strong correlations between organic acid metabolism and AmB accumulation. Overall, the organic acid analysis provided important insights into the potential factors of improving AmB production. To sum up, the CF strategy was favorable to supply abundant precursors and key intermediate metabolites for AmB biosynthesis.

## Conclusion

The effects of the key precursors and the differential metabolites on the production of amphotericin B by *S. nodosus* ZJB2016050 were investigated, and the combined addition of 4 mg/L isopropanol, 1 mM alanine, 1 g/L pyruvate, and 0.025 g/L nicotinamide was determined as the most effective additive feeding strategy. Meanwhile, fermentation for 24 h was tested as the best addition time. It demonstrates that the supplementation of different additives is effective to facilitate AmB production through regulating the metabolite node and decreasing the AmA content by affecting the activity of the ER5 domain. Furthermore, the efficiency of AmB synthesis could be significantly enhanced by establishing the pH staged control, two-stage temperature control, and DO staged control strategy in batch fermentation. With respect to the feeding regulation strategies, four different fed-batch fermentation feeding strategies were constructed, of which the highest AmB titer attained at 15.78 g/L was by the CSFB strategy. Based on the above results, a new approach for enhancing AmB biosynthesis was provided by a combinatorial feeding strategy under the most suitable fermentation process regulation combined with optimal mixed additives, which successfully improved AmB production from 9.89 to 18.39 g/L, with 85.9% increase. To our best knowledge, remarkable differences in the concentrations of organic acids were revealed to further explain the influence of the different strategies on the metabolic process during fermentation for the first time. These strategies developed in this research could be further extended to titer improvement on other important polyene macrolide antibiotic products.

## Data Availability Statement

All datasets generated for this study are included in the article/Supplementary Material.

## Author Contributions

BZ, Y-HZ, Z-QL, and Y-GZ participated in designing of this work. The analysis and the interpretation of experimental data were jointly conducted by all authors. BZ and Y-HZ prepared the manuscript with the help of Z-QL. Y-HZ, YC, KC, S-XJ, and KH performed the experiments. All authors approved the submitted version.

## Conflict of Interest

The authors declare that the research was conducted in the absence of any commercial or financial relationships that could be construed as a potential conflict of interest.
