# Supplementary material for: Enhanced AmB Production in Streptomyces nodosus by Fermentation Regulation and Rational Combined Feeding Strategy
Source: Front Bioeng Biotechnol. 2020 Jul 15;8:597. doi: 10.3389/fbioe.2020.00597 (PMC7373727; doi:10.3389/fbioe.2020.00597)
Supplement: Supplementary file 1 [file Data_Sheet_1.pdf]

Supplementary Material

1 Supplementary Tables

Table S1 AmB titer and AmA concentration of *Streptomyces nodosus* in an orthogonal L32 experiments

Run	X_1[a] (mg/L)	X_2[b] (mmol/L)	X_3[c] (mmol/L)	X_4[d] (%)	X_5[e] (%)	X_6[f] (%)	AmB titer (g/L)	Ratio of AmA (%)
1	0	0	0	0	0	0	5.160	3.05%
2	0	0	0	0	0.1	0.0025	5.261	1.17%
3	0	0	0	0.0025	0	0.0025	5.342	3.44%
4	0	0	0	0.0025	0.1	0	4.603	0.35%
5	0	0	1	0	0	0.0025	5.354	3.05%
6	0	0	1	0	0.1	0	5.572	4.16%
7	0	0	1	0.0025	0	0	5.198	5.14%
8	0	0	1	0.0025	0.1	0.0025	5.530	3.58%
9	0	4	0	0	0	0.0025	4.378	4.50%
10	0	4	0	0	0.1	0.0025	5.118	4.72%
11	0	4	0	0.0025	0	0	5.834	2.84%
12	0	4	0	0.0025	0.1	0.0025	5.468	1.89%
13	0	4	1	0	0	0	5.985	2.53%
14	0	4	1	0	0.1	0.0025	5.349	1.41%
15	0	4	1	0.0025	0	0.0025	5.278	1.74%
16	0	4	1	0.0025	0.1	0	6.090	1.49%
17	4	0	0	0	0	0.0025	4.524	4.20%
18	4	0	0	0	0.1	0	4.771	3.60%
19	4	0	0	0.0025	0	0	5.014	3.71%
20	4	0	0	0.0025	0.1	0.0025	5.113	3.43%
21	4	0	1	0	0	0	5.160	4.36%
***22**	**4**	**0**	**1**	**0**	**0.1**	**0.0025**	**6.632**	**2.47%**
23	4	0	1	0.0025	0	0.0025	5.568	1.92%

24	4	0	1	0.0025	0.1	0	5.763	1.51%
25	4	4	0	0	0	0	5.185	2.69%
26	4	4	0	0	0.1	0.0025	4.996	1.11%
27	4	4	0	0.0025	0	0.0025	5.202	1.99%
28	4	4	0	0.0025	0.1	0	4.896	1.41%
29	4	4	1	0	0	0.0025	4.889	2.57%
30	4	4	1	0	0.1	0	5.119	1.50%
31	4	4	1	0.0025	0	0	5.153	2.98%
32	4	4	1	0.0025	0.1	0.0025	5.182	2.24%

[a] X_1: Isopropanol; [b] X_2: Serine; [c] X_3: Alanine; [d] X_4: D-calcium pantothenate; [e] X_5: Pyruvate; [f] X_6: Nicotinamide

Table S2 Effects of different stirring speed on biomass, AmA content, AmB titer and productivity

Agitation rates (rpm)	PMV (%)	Ratio of AmA (%)	Fermentation cycle (h)	Production of AmB (g/L)	Productivity of AmB [g/(L·h)]
400	38.5	2.25	144	9.82	0.068
500	36	1.24	144	10.12	0.070
600	32	6.78	144	10.06	0.070

Table S3 Effects of different initial glucose concentrations on biomass, AmA content, AmB titer and productivity

Initial glucose concentration (g/L)	PMV (%)	Ratio of AmA (%)	Fermentation cycle (h)	Production of AmB (g/L)	Productivity of AmB [g/(L·h)]
50	21	0.21	120	7.57	0.063
60	26	0.94	120	7.83	0.065
70	34	2.98	120	9.89	0.083
80	32	4.03	120	7.77	0.065

Table S4 Extracellular organic acids concentration with different pH control strategies at different timepoints: 48, 72, 108 and 144

Organic acids	48 h			72 h			108 h			144 h		
	pH 6.7	pH 7.0	pH 7.0-6.7	pH 6.7	pH 7.0	pH 7.0-6.7	pH 6.7	pH 7.0	pH 7.0-6.7	pH 6.7	pH 7.0	pH 7.0-6.7
oxalic acid (Ox)	<0.1	0.5±0.2	<0.1	<0.1	0.5±0.1	<0.1	<0.1	0.5±0.1	<0.1	<0.1	0.5±0.2	<0.1
α-ketoglutaric acid (α-KG)	0.9±0.2	1.3±0.4	<0.1	1.5±0.3	1.5±0.3	0.5±0.1	1.5±0.2	1.8±0.2	0.1±0	0.5±0.1	1.9±0.5	<0.1
pyruvic acid (Py)	1.4±0.3	0.4±0.1	<0.1	2.8±0.4	0.3±0	<0.1	2.6±0.3	0.2±0	<0.1	0.7±0.1	<0.1	<0.1
formic acid (FA)	0.2±0	0.4±0	<0.1	0.7±0.2	0.6±0.1	<0.1	1.2±0.3	0.9±0.2	0.1±0	0.3±0	1.2±0.3	<0.1
acetic acid (Ac)	<0.1	<0.1	0.7±0.2	<0.1	<0.1	3.0±0.8	2.3±0.3	2.9±0.5	8.4±1.2	3.7±0.7	<0.1	12.7±1.5
Lactic acid (Lac)	0.7±0.1	<0.1	0.5±0.1	<0.1	<0.1	0.5±0.2	0.4±0	<0.1	0.4±0.1	<0.1	<0.1	0.3±0
Citric acid (Cit)	0.8±0.2	1.3±0.2	<0.1	1.5±0.3	2.0±0.5	2.0±0.2	2.3±0.5	2.2±0.3	3.1±0.6	1.1±0.3	3.2±0.6	0.1±0

The values shown here represent the means of three independent experiments and the error bars represent standard deviations of three values. The concentration of the metabolites was given in g/L.

Table S5 Extracellular organic acids concentration with different temperature control strategies at different timepoints: 48, 72, 108 and 144 h

Organic acids	48 h			72 h			108 h			144 h		
	26 °C	30 °C	30 - 26 °C	26 °C	30 °C	30 - 26 °C	26 °C	30 °C	30 - 26 °C	26 °C	30 °C	30 - 26 °C
oxalic acid (Ox)	<0.1	0.4±0.1	0.5±0.2	0.4±0.2	0.5±0.1	0.6±0.1	0.5±0	0.8±0.2	0.8±0.1	0.8±0.1	0.8±0.2	0.6±0.1
α-ketoglutaric acid (α-KG)	0.8±0.1	1.8±0.3	2.5±0.8	2.2±0.2	3.6±0.8	4.0±0.6	4.0±0.4	5.7±0.9	4.2±0.5	10.2±0.5	6.3±0.4	4.0±0.3
pyruvic acid (Py)	0.7±0.2	1.2±0.1	1.6±0.3	1.2±0.4	1.7±0.25	1.7±0.2	1.5±0.2	0.9±0.1	0.9±0.1	1.6±0.2	0.8±0.2	0.6±0.1
formic acid (FA)	0.1±0	0.2±0	0.3±0.1	<0.1	<0.1	0.8±0	0.3±0.1	0.7±0.3	<0.1	<0.1	0.9±0.1	<0.1
acetic acid (Ac)	<0.1	<0.1	0.8±0.1	<0.1	<0.1	<0.1	<0.1	<0.1	0.7±0.1	5.3±0.9	5.3±0.9	3.0±0.6
malonate (Mal)	0.4±0	0.9±0.1	2.1±0.6	0.7±0.2	1.7±0.1	3.4±0.3	0.9±0.2	4.3±0.9	5.1±0.5	2.2±0.3	3.6±0.4	4.2±0.8
succinic acid (Suc)	0.1±0	0.1±0	0.1±0	0.4±0.1	<0.1	0.7±0.1	0.11±0	<0.1	1.6±0.2	<0.1	<0.1	0.4±0

The values shown here represent the means of three independent experiments and the error bars represent standard deviations of three values. The concentration of the metabolites was given in g/L.

Table S6 Extracellular organic acids concentration with different dissolved oxygen control strategies at different timepoints: 48, 72 ,108 and 144 h

Organic acids	48 h			72 h			108 h			144 h		
	20%	30%	40%	20%	30%	40%	20%	30%	40%	20%	30%	40%
oxalic acid (Ox)	<0.1	<0.1	<0.1	0.4±0.2	<0.1	<0.1	0.5±0	0.1±0	<0.1	0.8±0.1	0.2±0	<0.1
α-ketoglutaric acid (α-KG)	1.2±0.2	1.1±0.3	0.6±0.1	2.8±0.4	1.7±0.6	2.1±0.4	4.3±0.9	3.1±0.8	2.4±0.3	4.7±0.6	3.0±0.9	2.4±0.3
pyruvic acid (Py)	1.3±0.1	1.4±0.4	1.4±0.2	1.6±0.3	1.7±0.3	2.2±0.3	1.0±0.1	0.7±0.2	0.7±0.2	0.1±0	0.7±0.1	0.2±0
formic acid (FA)	0.2±0	<0.1	<0.1	0.8±0.2	0.1±0	<0.1	0.2±0	0.2±0	0.6±0.1	1.7±0.4	0.2±0	0.2±0
acetic acid (Ac)	<0.1	<0.1	<0.1	0.1±0	<0.1	<0.1	<0.1	0.1±0	0.5±0.1	0.1±0	0.6±0.1	<0.1
Lactic acid (Lac)	0.4±0.1	<0.1	<0.1	0.8±0.3	<0.1	0.4±0.1	0.5±0.1	<0.1	1.0±0.1	0.7±0.2	<0.1	0.7±0.1
Citric acid (Cit)	1.1±0.3	1.1±0.2	1.2±0.3	1.1±0.2	1.2±0.4	1.1±0.2	1.1±0.1	0.8±0.1	2.5±0.6	2.5±0.5	<0.1	3.1±0.8

The values shown here represent the means of three independent experiments and the error bars represent standard deviations of three values. The concentration of the metabolites was given in g/L.

Table S7 Extracellular organic acids concentration under different fed-batch strategies at different timepoints: 48, 96 ,120 and 156 h

Organic acids	48 h			96 h			120 h			156 h		
	CSFB	CGFB	CF	CSFB	CGFB	CF	CSFB	CGFB	CF	CSFB	CGFB	CF
oxalic acid (Ox)	<0.1	<0.1	<0.1	<0.1	0.5±0	<0.1	<0.1	0.6±0.1	<0.1	<0.1	0.7±0.3	<0.1
α-ketoglutaric acid (α-KG)	1.7±0.3	2.0±0.5	0.4±0	2.7±0.6	3.5±0.9	0.4±0	3.3±0.7	3.3±0.2	<0.1	3.7±0.5	4.0±0.8	<0.1
pyruvic acid (Py)	0.8±0.1	1.1±0.2	1.1±0.3	0.6±0	0.8±0.1	0.6±0.1	1.4±0.3	1.2±0.3	0.4±0	1.6±0.4	1.8±0.5	0.1±0
formic acid (FA)	<0.1	<0.1	0.1±0	0.2±0	<0.1	0.4±0	0.8±0.1	<0.1	0.6±0.1	0.3±0	<0.1	0.9±0.2
acetic acid (Ac)	<0.1	1.3±0.2	<0.1	<0.1	3.7±0.2	0.1±0	0.6±0.1	1.5±0.2	4.1±0.5	1.0±0.1	3.5±0.9	7.4±0.9
Lactic acid (Lac)	0.5±0	1.7±0.4	0.4±0	0.7±0.1	3.0±0.5	1.1±0.2	0.9±0.3	2.9±0.8	0.8±0.2	1.0±0.2	5.2±0.9	1.0±0.2
Citric acid (Cit)	0.6±0.1	<0.1	1.5±0.2	0.5±0	1.0±0.2	0.7±0.2	0.8±0.1	2.9±0.6	0.9±0.1	2.8±0.3	1.3±0.2	3.8±0.5

The values shown here represent the means of three independent experiments and the error bars represent standard deviations of three values. The concentration of the metabolites was given in g/L

2 Supplementary Figures

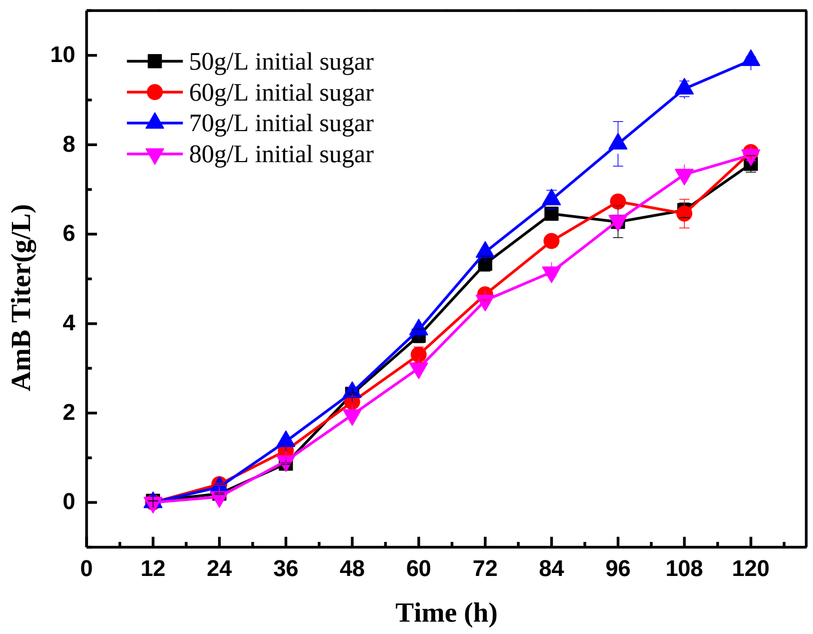

Fig S1. AmB production at different initial glucose concentrations

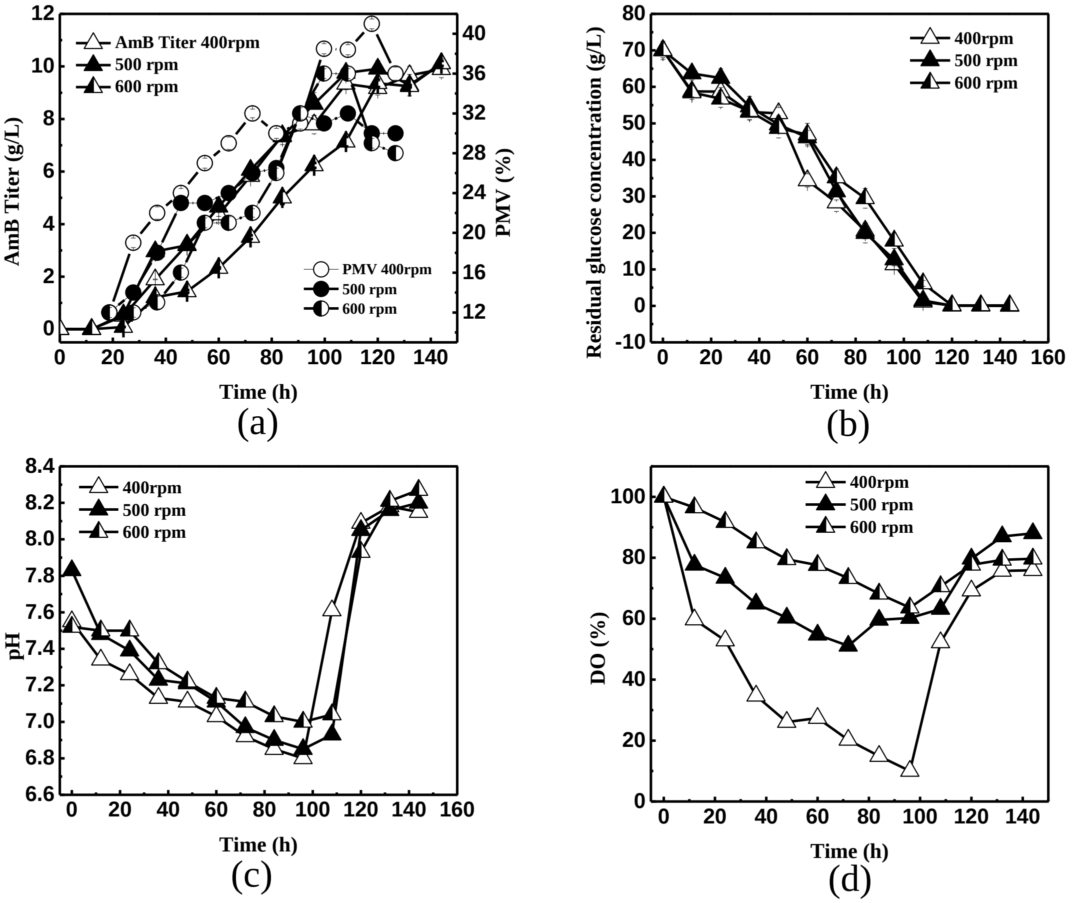

Fig S2. The effect of different agitation rates on AmB production during batch cultivation in 5-L fermentor. The culture temperature was 26 °C, and the medium pH was in a natural status. (a) AmB biosynthesis and cell growth; (b) glucose consumption; (c) pH range; (d) DO range. Data are the average of three batch cultures. For clarity, error bars are not displayed.

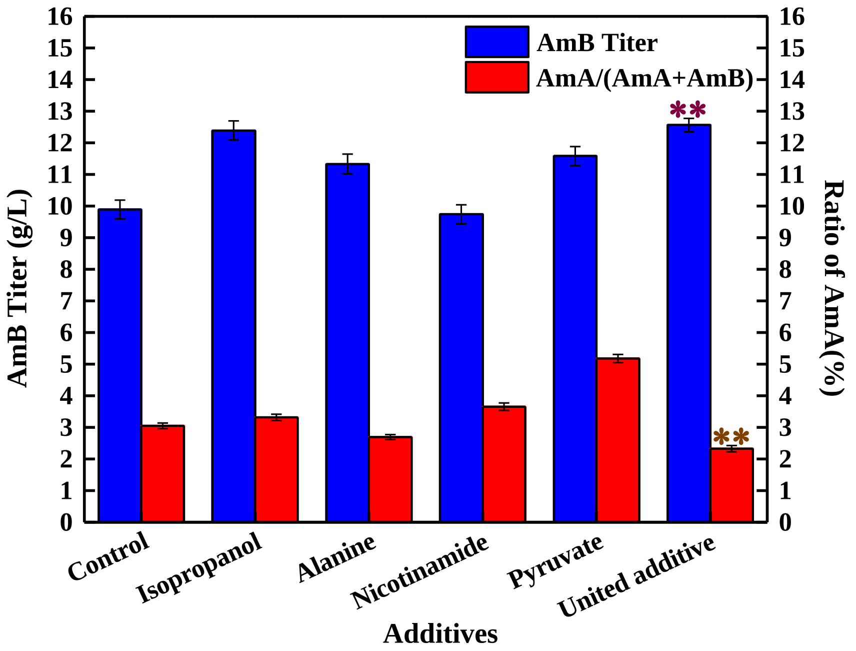

Fig S3. Effects of different addition strategies on the production of AmA and AmB, including separate addition of isopropanol, alanine, nicotinamide, pyruvate and the supplementation of united additives in 5-L fermentor. "**" symbols represent the optimum additive feeding strategy. The values shown represent the means of three independent experiments and the error bars represent standard deviations of three values.
